# Enabling Clustering for Privacy-Aware Data Dissemination Based on Medical Healthcare-IoTs (MH-IoTs) for Wireless Body Area Network

**DOI:** 10.1155/2020/8824907

**Published:** 2020-11-28

**Authors:** Fasee Ullah, Izhar Ullah, Atif Khan, M. Irfan Uddin, Hashem Alyami, Wael Alosaimi

**Affiliations:** ^1^Department of Computer and Information Science, Faculty of Science and Technology, University of Macau, Macau, China; ^2^Institute of Business and Management Sciences, Peshawar, KP, Pakistan; ^3^Department of Computer Science, Islamia College, Peshawar, KP, Pakistan; ^4^Institute of Computing, Kohat University of Science and Technology, Kohat, Pakistan; ^5^Department of Computer Science, College of Computers and Information Technology, Taif University, P.O. Box 11099, Taif 21944, Saudi Arabia; ^6^Department of Information Technology, College of Computers and Information Technology, Taif University, P.O. Box 11099, Taif 21944, Saudi Arabia

## Abstract

There is a need to develop an effective data preservation scheme with minimal information loss when the patient's data are shared in public interest for different research activities. Prior studies have devised different approaches for data preservation in healthcare domains; however, there is still room for improvement in the design of an elegant data preservation approach. With that motivation behind, this study has proposed a medical healthcare-IoTs-based infrastructure with restricted access. The infrastructure comprises two algorithms. The first algorithm protects the sensitivity information of a patient with quantifying minimum information loss during the anonymization process. The algorithm has also designed the access polices comprising the public access, doctor access, and the nurse access, to access the sensitivity information of a patient based on the clustering concept. The second suggested algorithm is *K*-anonymity privacy preservation based on local coding, which is based on cell suppression. This algorithm utilizes a mapping method to classify the data into different regions in such a manner that the data of the same group are placed in the same region. The benefit of using local coding is to restrict third-party users, such as doctors and nurses, when trying to insert incorrect values in order to access real patient data. Efficiency of the proposed algorithm is evaluated against the state-of-the-art algorithm by performing extensive simulations. Simulation results demonstrate benefits of the proposed algorithms in terms of efficient cluster formation in minimum time, minimum information loss, and execution time for data dissemination.

## 1. Introduction

**Figure 1:**
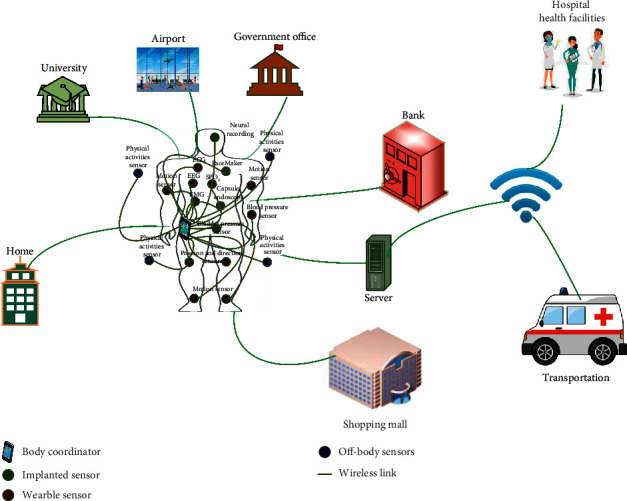
Medical healthcare-IoT-based patient's health monitoring and data dissemination.

The wireless body area network (WBAN) for patient health monitoring is a leading technology of the current decade. Different biomedical sensors (BMSs) are used in WBAN to monitor the patient's vital signs. The vital signs of patient are heart beat rate, respiratory rate, blood pressure, and temperature [1]. Moreover, different BMSs are installed on the patient's body and inside the patient's body, and some BMSs are placed around the patient's body to monitor different physical activities. These BMSs monitor patient's vital signs, and the monitored data are transmitted to the body coordinator (centralized node), which is responsible to immediately transmit all the patient's health information to the physicians in real time, and if an emergency situation is detected, the physician will instantly inform the patient through the computer system by sending suitable messages or alarms. The whole scenario is implemented on the Medical Healthcare IoT, as shown in Figure 1. The data transmission is categorized into three phases. The first phase collects the sensory data using a centralized node. In the second stage, the centralized node forwards the sensory data to the base station. The base station transmits the received data to the medical staff, in the third phase. Moreover, this whole network is established via IoT network-based concepts [2]. The transmission contains reading of the sensory data and other details such as patient's name, disease, zip code, and age. There is a possibility of the data privacy issue with patient's data for sharing and processing with a doctor or nurse who may see all the information. In addition, the patient data may be stolen between the three phases in the transmission process. Thus, there is a need to design an efficient and secure data privacy technique based on machine learning when data are at risk of being stolen.

In this work, the *K*-medoid machine learning algorithm is used for clusters formation and local recording algorithm is employed for data privacy. In local recoding, there is a less chance of information loss during data transmission. Prior studies have also presented data privacy algorithms based on clustering such *K* member and OKA [3]. However, it has been noted that these algorithms take an enormous amount of time in cluster formation and suffer from information loss. In literature, major research has used anonymity, data masking, and data padding for data privacy [4, 5]. These techniques have issues of privacy, standardization, digital forgetting, mobility, object name servers (ONSs), naming, traffic characterization, quality support, data integrity, and authentication [6]. These issues are NP hard problems in the existing privacy algorithms, and most of them have considered issues of incognito, clustering, global recoding, and diversity in privacy. In addition, cluster mechanisms and local coding techniques have a greater impact on the privacy of data in the healthcare sector. The personal information privacy is the most important aspect. In privacy leakage, the invader can use any information for diverse purpose. Thus, this work has focused on the challenging issues of data privacy when data are communicated through IoT devices because any physical entity or object can be compromised. Majority of devices and sensors are operated through batteries in IoT environment with small battery and little processing energy consumption compared to data privacy being the major concern.

In this perspective, this paper proposes a clustering mechanism for privacy-aware data dissemination based on medical healthcare-IoTs (MH-IoTs) for wireless body area network. The proposed mechanism is compared with different machine learning algorithms using standard datasets for patient's data privacy when a medical doctor reviews her health report. Specially, the design of the proposed mechanisms is broadly divided into three folds:

- To propose an algorithm based on the clustering technique (*K*-medoid), to enumerate the information loss at the anonymization process, with the aim of reducing the information loss, and to preserve the personal identification in healthcare domain.
- To design a *K*-anonymity model for privacy preserving using local recoding, which is based on cell suppression. The local recoding algorithm utilizes a mapping method to classify the data into different regions and places the same kind of data into the same region. Thus, the strength of using local recoding is to restrict the third-party users such as doctors and nurses when trying to insert incorrect values in order to access real patient data.
- Extensive simulations are carried out to assess the performances of the existing machine learning-based algorithms in terms of information loss and execution time for cluster formation and data privacy.

The rest of the work is arranged in following manner: Section 2 presents the existing work.  Section 3 introduces the proposed clustering and *K*-anonymity-based data privacy mechanism (using local recording).  Section 4 addresses the empirical assessment of the proposed algorithm against state-of-the-art algorithm.  Section 5 concludes the research work.

## 2. Related Work

Numerous studies have been presented on patient data privacy based on the medical healthcare-IoTs (MH-IoTs) infrastructure. This paper [7] has focused on data privacy issues in social networks. The study also highlighted the privacy issues of the nodes deployed in the networks. In addition, the top-down approach, based on *K*-anonymization, has been adopted to protect privacy for individuals and organizations [8]. All information is stored in graph nodes, and the similarity index between the two nodes represents the weight of the edges in graph. The study [9] showed that the *K*-anonymization is a NP hard problem and proposed an algorithm, known as graph-based local recording for data anonymization. They adopted real and synthetic datasets for the simulations for *K*-anonymity problems [10]. On the contrary, these simulations have opened up security challenges in a distributed environment for IoT to know how to handle the Internet in the future. The existing Internet is connected to different types of nodes, such as sensors, systems, software, and applications. They work together in a single environment, known as IoT. However, the issue of data privacy in a diverse environment is a challenge. For this purpose, this study [11] has developed the cooperative distributed systems (CDSs) by connecting with the contract net protocol (CNP).

Existing studies have described security vulnerabilities of the operation layer in IoT, such as network layers, physical layers, application layers, and processing layers. [12]. IoT is essential in our lives to take effective action to protect the privacy/confidentiality and security of the user. [13]. The study in [14] has tried to establish anonymity in IoT networks by classifying the networks into coverage and the technologies deployed in IoT. Through these classifications, it is easy to get information of sources. However, Lopez et al. [15] have observed that the study in [14] has not considered the privacy risk. Alabdulatif et al. [16] have explored the aim of creating a health surveillance system, making health groups smarter with the goal of detecting frequent variations in the patient's signs, elders living in assisted living surroundings, and healthy people living in smart home. The patient's data privacy problem has been handled with cloud computing by sharing the reading of vital signs to medical staff via the protected IoT environment. The narrative-based anonymity model has been developed, known as PPDC, and has been implemented on the client-server model to take care of anonymous records with reduction of privacy issues [17]. Luo et al. [18] have inherited the basic merits in [11] by developing a scheme, known as the secret sharing scheme (SW-SSS) with optimization of the secret share and exactly repairs of the shared data with storage of the patient's data in cloud. This study also claims that the patient data are safe if a server is comprised. The study [19] has evaluated and identified the privacy means, tradeoff between privacy, efficiency, and quality of the model. Zhou et al. [20] investigated the data privacy problem caused when there is increase in operators and by the approval of IoT technologies. Moreover, the framework was settled to inspect and find the consequences of privacy and security in provision of IoT new technologies [21]. This paper [22] has developed a chaos-based encryption model for a patient's data privacy. The patient data are encrypted in form of image with casualness behaviour which ensures efficiency and the uppermost level of safekeeping from counter attacks. The electrocardio graph (ECG) for IoT-based medical care was developed for validation by removing the noises with the privacy protection [23]. To improve the IoT environment for data privacy, the fog computing environment has been embedded for fast response with low latency [24]. However, the implementation of fog computing has improved the connectivity problems, but there is a need to efficiently handle data privacy in fog-based IoT deployment. The patient's revocation has been suggested in [25] with the core concept implementation of block chain technology for healthcare. Moreover, the smart cross-domain data sharing, self-adaptive access control, and smart deduplication supports have been introduced for data privacy, sharing in restricted mode and a user revocation [26]. The authors in [27] suggested EETP-MAC protocol to transmit the patient's data using prioritization by classifying into different perceptions with consideration of reduced energy consumption. The efficient design of MAC superframe structure is presented in [28] for controlling the nonemergency data with enhanced performances. However, these papers have not considered handling the patients' data from multiple environments. The anonymization problem has validated through mathematical testing for cluster formation and information exchange [29, 30]. Thus, the existing studies on the data privacy of patient's health monitoring-based IoT dissemination have motivated to design and develop efficient mechanisms with required minimum time for data transmission with high data privacy.

## 3. The Proposed Work

### 3.1. Overview

There are various BMSs deployed to monitor vital signs of patients, and these BMSs have connected in STAR/MESH topology to the body coordinator. The aim of this proposed work is to protect the privacy of patient's data such as name, disease, age, gender, and zip code from medical doctor, public, and paramedic staff. Hence, this study has proposed two schemes that are cluster-based privacy preserving and local recoding using *K*-anonymity model for privacy preserving, which are explained below.

### 3.2. Cluster-Based Technique for Privacy Preserving

**Figure 2:**
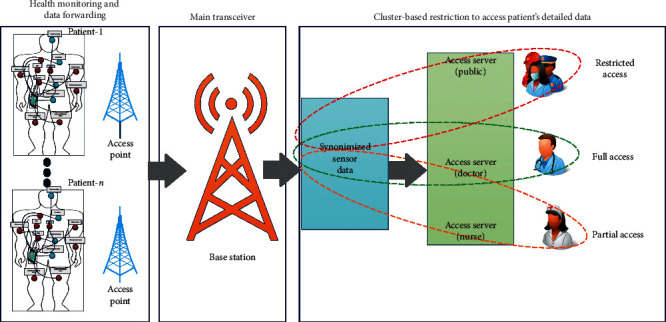
Proposed architecture for data privacy preserving based on cluster.

The most efficient way of the resource allocation with certain restricted conditions among public, doctor, and medical staff has been acquired via the cluster technique, as depicted in Figure 2. The cluster divides the large spaces into *n* spaces and allocates the private spaces according to the policies. The advantage of the cluster technique is to efficiently manage the patient's data with access policies, and through this way, privacy preserving is achieved by losing minimum information. Figure 2 shows a clustering technique by classifying health monitoring and data forwarding, main transceiver, and cluster-based restriction to access patient's data. The health monitoring and data forwarding monitor health of *m* patients, and the monitored data are then forwarded to the body coordinator. Moreover, the body coordinator sends the monitored data to access points wherein the access points forward data to the base station. The most important stage is the cluster-based restriction to access patient's data that have been achieved via the anonymity technique. The anonymity technique is based on the machine learning approach and is known as *K*-medoid.

The working procedure of *K*-medoid machine learning algorithm is to identity the nearest objects in the whole data elements and assigns the identified objects to the same cluster of data elements. The identified objects of the same cluster of the neighbours are assigned to groups to the anonymity algorithm. The anonymity algorithm is based on *K*-medoid which is implemented on the cluster technique and allows to access the patient's data according to the group and identity of personnel. Through this implementation, all the processes are executed in short time.

*K*-medoid is a partitioning algorithm that splits the *n* data points of dataset into *K*-nonoverlapping predefined distinctive groups known as clusters, where each data point goes into a single cluster. It is more robust and less prone to noise and outliers as compared to the *K*-means clustering algorithm. Medoids (actual points) are used as cluster centers instead of *K*-means average points. In addition, it is simple and converges in a certain number of steps. *K*-medoid identifies each cluster with a single data point inside known as medoid of a cluster. It is also known as partitioning around medoid (PAM). The term medoid is the point inside a cluster whose average similarity with other data points in the cluster is greater. Aim of *K*-medoid algorithm is to minimize the dissimilarities summation among cluster medoid and data point in a cluster, as depicted in Algorithm 1. *K*-medoid cost is given as follows:

(1) $$\begin{array}{c} \underset{P_{i}\in C_{i}}{\sum }\underset{P_{i}\in C_{i}}{\sum }\left|P_{i}-C_{i}\right|. \end{array}$$

### 3.3. Local Recoding-Based *K*-Anonymity Model for Privacy Preserving

The proposed *K*-anonymity model of local recoding for privacy preserving algorithm is based on cell suppression. This algorithm classifies data into different regions and places the classified data to the same region of the same group via a mapping method. Thus, the benefit of using local recoding is to restrict the third parity users like doctors and nurses when they try to insert wrong values for accessing the real data of patients.

**Figure 3:**
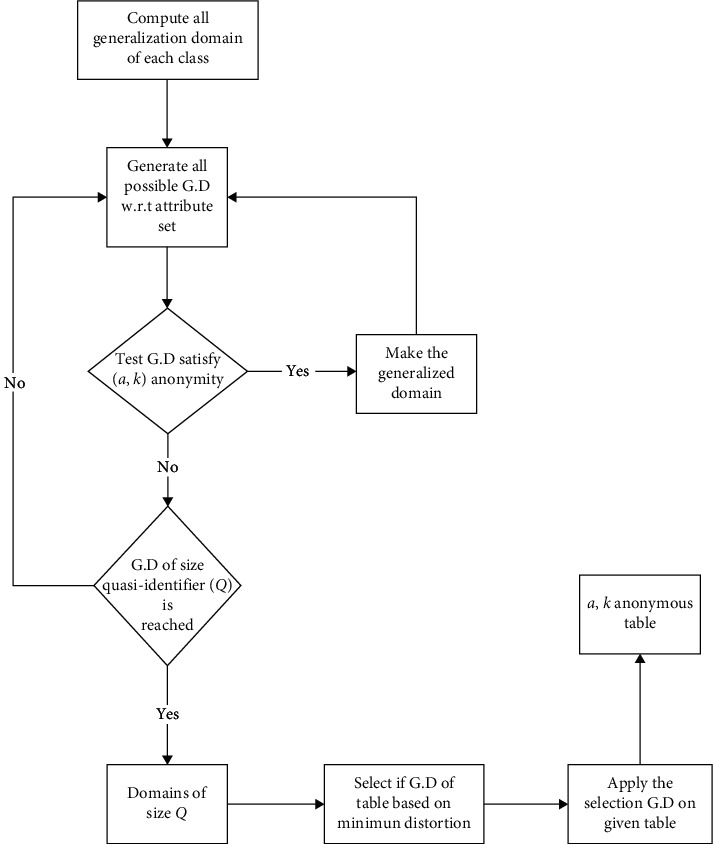
Local recoding-based anonymity.

The proposed algorithm 2 for local recoding is presented. First, this algorithm computes all generalized domain (GD) of each class. Subsequently, the second step indicates to compute again all possible attributes of GD (e.g., G0 and B0). After, the generation of all generalized domains is verified whether GD is *a,k* anonymous. If it is true, then the generalization domain is selected. However, this process will repeat and verify all GD until an appropriate selection is achieved (e.g., size of quasi). Continuing in the steps, if the size of quasi-identifier (*Q*) is reached to threshold values, then it selects GD table based on distortion. Otherwise, it will go again to the second step to compute all possible attributes of GD. Finally, the achieved generalization domain data are applied to table by achieving an *a*, *k*-anonymity.  Figure 3 shows the flowchart of the proposed local recoding algorithm.

## 4. Performance Evaluation

This section explains the performance simulation of the proposed work and has been compared with the state-of-the-art works. It is divided into three sections. First section describes the simulation environment. Second section describes the performance evaluation metrics which have used in performance measurement of the proposed work and compared with state-of-the-art works. Thirdly, the results of the proposed algorithms have been evaluated and discussed in phase 1, while the phase 2 represents the performance comparison of the proposed algorithms with the state-of-the-art algorithms.

### 4.1. Simulation Environment

The simulation environment has been setup on Dell machine having a CPU speed of 2.4 GH along with 6 GB RAM. Microsoft windows 10 operating system was used, and the simulation has been carried out in Python Anaconda Spyder v2.7. This simulation has used datasets of UC Irvine Machine Learning repository. Moreover, the datasets contain numerical and categorical data elements of 14 attributes with 48842 instances. There are nine attributes chosen for *K*-anonymity comprising gender, marital status, education, occupation, race, and native country as a quasi-identifier. Age, education, and salary are treated numerical attributes, while the others categorical.

### 4.2. Simulation Performance Metrics

The selected simulation performance metrics have reduced information loss and increased execution efficiency, as explained in detail.

#### 4.2.1. Reduction in Information Loss

The attributes in table contain numeric and categorical values with minimum and maximum distances of the *X* generalized equivalence classes. Thus, the information loss (*LS*) for numerical attributes can be expressed as follows:

(2) $$\begin{array}{c} LS=\frac{{\text{Max}}_{x}-{\text{Min}}_{x}}{\text{Max}-\text{Min}}. \end{array}$$

The information loss for categorical attributes is represented in tree with *n* levels, as follows:

(3) $$\begin{array}{c} LS=\frac{S*n}{S}, \end{array}$$

(4) $$\begin{array}{c} LS=j=\sum_{i}^{n}\frac{LSj}{n}, \end{array}$$

where *S* is denoted by the size of the attribute. The execution performance is improved.

To compute the system efficiency and analyse the time consumption of the local recoding algorithm in processing patient data via BMSs, the number of suppression and generalization procedures is incorporated to get local recording and cluster-based anonymity.

### 4.3. Analysis of Results and Discussion

There are three parameters considered to measure the performance of the proposed work compared with state-of-the-art works that are information loss, execution time, and number of cluster (*k*).

#### 4.3.1. Information Loss in *K*-Medoid Based on *K*

**Figure 4:**
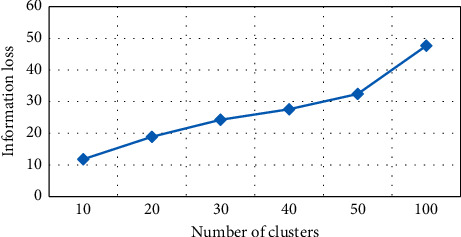
Number of clusters vs information loss in *K*-medoid.

Information loss based on *K*-medoid algorithm has been tested for number of clusters (*K*), as shown in Figure 4. For every value, the graph will have different grounds on *K* values and information loss. For instance, if *K* = 10, information loss is 11.24% of the anonymity algorithm. It depicts *K* values, and information loss is directly related. Decreasing the *K* will automatically result in decreasing information loss. The mentioned graph is created from six experimental values of *K*, i.e., 10, 20, 30, 40, 50, and 100 with corresponding information loss. The NCP value is 11.24% at *K* = 10, and the value is 15.03% at *K* = 20, as highlighted in Table 1, showing various values of *K* and NCP in information loss.

#### 4.3.2. Execution Time in Anonymity Based on *K*

**Figure 5:**
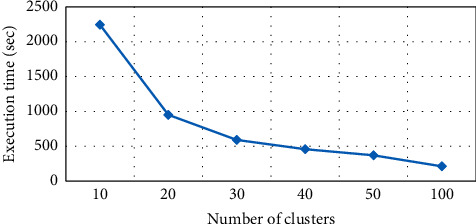
Number of clusters vs execution time in *K*-medoid.

Performance of anonymity algorithm has been presented in terms of *K* values verses time, as shown in Figure 5. Changing the value of *K* results in varied time. For each value, the performance will be changed. At *K* = 10, the anonymity algorithm will spend 1.42 seconds, and 0.93 seconds will be spent for the *K* value of 20. Therefore, *K* and time are indirectly related to each other. The presented results are taken from six experimental values of *K*, i.e., 10, 20, 30, 40, and 50. Some minor variation occurs at *K* = 30 that spends 0.82 seconds.  Table 2 presents statistics analysis of number of clusters versus execution time.

#### 4.3.3. Information Loss and Execution Time of OKA (One-Pass *K*-Means Algorithm) Based on *K*

**Figure 6:**
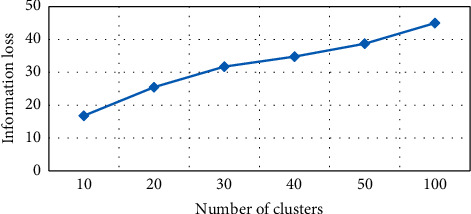
Number of clusters vs NCP in OKA.

**Figure 7:**
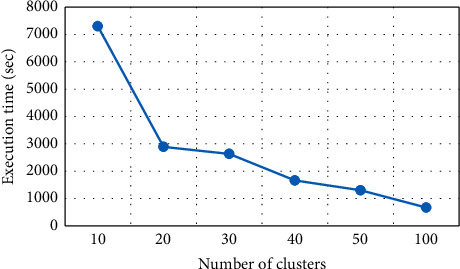
Number of clusters vs execution time of OKA.

Information loss of the algorithm is 16.78% when the value *K* is set as 10, and at *K* = 20, the resultant NCP is 25.47%, as shown in Figure 6. These NCP values gradually go up as more *K* values are added. Increasing the value of *K* will result in increasing information loss and vice versa that unfolds that both *K* values and information loss are directly related. The presented results are picked up from six experimental values of *K*, i.e., 10, 20, 30, 40, 50, and 100. In the same way, the OKA algorithm requires 7300.11 sec times for execution of 10 clusters (*K*), as shown in Figure 7. Here, also changing the value of *K* takes varied time that shows *K* and time are indirectly related.

#### 4.3.4. Execution Time and Information Loss of *K* Member Based on *K*

**Figure 8:**
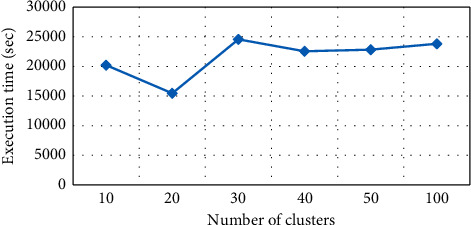
Number of clusters vs execution time in *K*-member.

**Figure 9:**
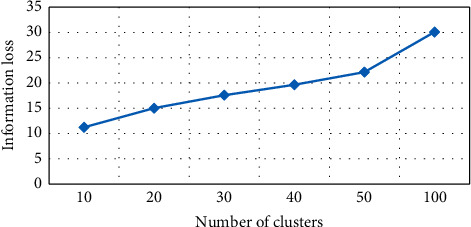
Number of clusters vs execution time in *K*-member.

The execution time performance has been compared based on values of *K*, as shown in Figure 8. To alter the value of *K*, change will occur in time although time will not continuously increase but depend on *K*.  Figure 9 shows the information loss of 11.24% at *K* = 10. Both *K* and information loss are directly related. The results presented in the graph are generated from six different experimental *K* values, i.e., 10, 20, 30, 40, 50, and 100. Change in the information loss is also noted. For instance, its value is 11.24% at *K* value 10 and 15.03% at *K* value 20.

### 4.4. Phase 2: Results Comparison of the Proposed Algorithms with State-of-the-Art Algorithms

#### 4.4.1. Execution Time of All Clusters Based on *K*

**Figure 10:**
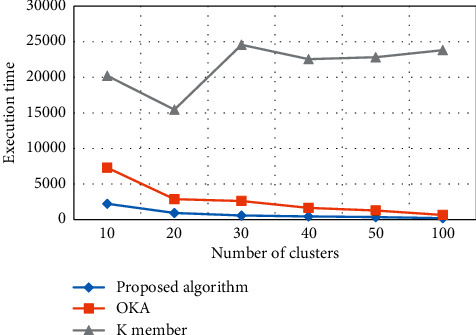
Number of different clusters vs execution time.

The performance of the proposed algorithm for cluster formation and the required execution time is compared with OKA and *K* member, as presented in Figure 10. When a cluster initial value is set 10, then the execution time for a cluster formation of *K* is very high as compared to OKA. In the same way, the proposed algorithm outperforms in terms of less time required for cluster formation when a cluster initial value is 10 as compared to OKA and *K* member. The reason is that the proposed algorithm selects a node with minimum value or distance as compared to both algorithms. The number of clusters along with NCP values is given in Table 3.

#### 4.4.2. Information Loss in Clusters Based on *K*

**Figure 11:**
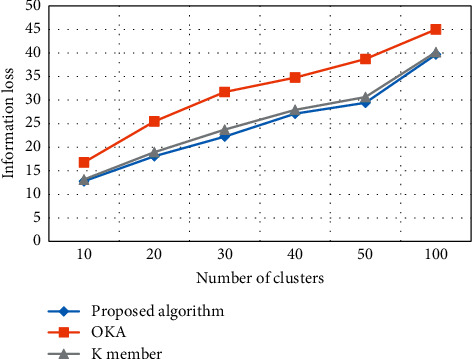
Information loss ratio in clusters.

The information loss of the proposed algorithm is compared with two clusters algorithms, that is, OKA algorithm and *K* member algorithm, as depicted in Figure 11. The information loss ratio is increased as the network extends in terms of further cluster formation. Thus, the performance of the proposed algorithm is comparatively better to OKA and *K* member. Performance measurement for information loss of OKA is higher than *K* member because *K* member thoroughly verifies the existing nodes in clusters caused by more information loss comparatively. Moreover, there is increase in information loss if *k* unceasingly alters which means the more the number of clusters, the more the information loss. Conversely, decreasing the number of clusters will result in decreasing value of information loss. The proposed algorithm has features of clusters set in the network. These clusters lessen the execution by adopting the substitute channels for providing the stream of bits from one end to another in the network. Inserting multiple clusters can condense the information loss during transmission due to incessant data monitoring by each cluster.

Time complexity is the central point to present proficient results. OKA takes *O*(*n*^2^/*K*), and *k* takes *O*(*n*^2^).The entire time complexity of the proposed algorithm is *O*(*nk* + *nd*), where *O*(d) specifies calculating a distance to one example. O(*nd*) reveals to compute the distance to every cluster. *O*(*nk*) is the entire time to find out the closest cluster in *k*.

## 5. Conclusion

The cluster-based resources sharing using machine learning approaches is one of the prominent concepts in WBAN. The first proposed algorithm is employed for preserving personal identification using cluster concepts with polices of the access restrictions. This algorithm has been efficiently performed for preserving personal identification in data sharing and information gathering in clusters. Also, the maximum number of clusters formation has consumed optimal time as compared to the existing machine learning algorithms. The second proposed algorithm has lost minimum information comparatively in OKA and *K* member algorithms. These performances significantly increase the privacy of the patient's data in a better way.

In the future, the proposed algorithm will improve and compare with a deep learning algorithm for the *K*-anonymity problem for patient's data privacy.

## Figures and Tables

**Algorithm 1:**
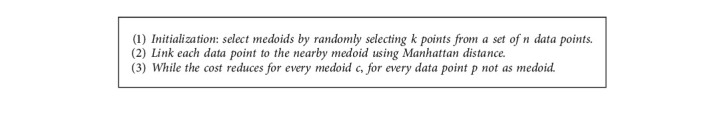
Pseudocode for *K*-medoid algorithm.

**Algorithm 2:**
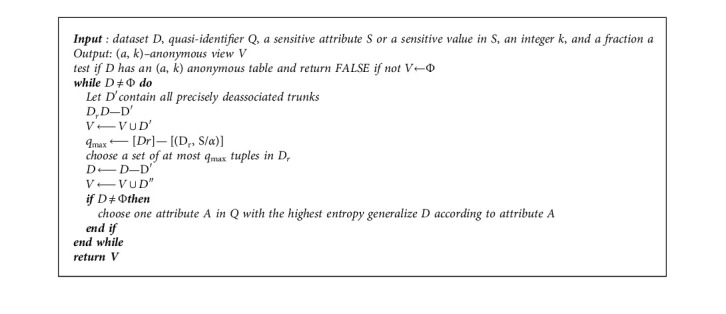
Pseudocode for local recoding.

**Table 1:** Information loss in *K*-medoid algorithm.

*K*	NCP
10	11.79
20	18.88
30	24.26
40	27.58
50	32.44
100	47.67

**Table 2:** Execution time in *K*-medoid.

Number of clusters	Execution time (sec)
10	2242.87
20	950.25
30	591.36
40	458.01
50	370.42
100	211.93

**Table 3:** Information loss in OKA algorithm.

Number of clusters	NCP (%)
10	16.78
20	25.47
30	31.72
40	34.77
50	38.71
100	44.99

## Data Availability

The data can be obtained from the corresponding author upon reasonable request.
